# DEPLOYING IN-HOME SENSORS TO COMMUNITY-DWELLING OLDER ADULTS: ADOPTION OF TECHNOLOGY AND LESSONS LEARNED

**DOI:** 10.1093/geroni/igac059.2697

**Published:** 2022-12-20

**Authors:** Erin Robinson, Gashaye Melaku Tefera, Geunhye Park, Marjorie Skubic, Noah Marchal, Mihail Popescu, James Keller, Rantz Marilyn

**Affiliations:** University of Missouri, Columbia, Missouri, United States; University of Missouri, Columbia, Missouri, United States; University of Missouri, Columbia, Missouri, United States; University of Missouri, Columbia, Missouri, United States; University of Missouri, Columbia, Missouri, United States; University of Missouri, Columbia, Missouri, United States; University of Missouri, Columbia, Missouri, United States; University of Missouri, Columbia, Missouri, United States

## Abstract

In-home, sensor technologies can promote chronic disease self-management and independence among older adults. However, the translation of these technologies from assistive living/long-term care to community-dwelling older adults is lacking. This study aimed to tailor such technologies for private use by older adults, and gather feedback about adoption/interpretation of sensor-generated health information to make informed health decisions. Participants (Nf33; 72.7% female) aged 60+ (Mean=79.5) had three types of sensors installed: 1) depth sensors that track gait parameters and falls; 2) passive infrared motion sensors that track room activity; and 3) hydraulic bed mats that track sleep, respiration, and heart rate. Participants were also offered a Garmin smartwatch/fitness tracker. A health app was developed for participants to retrieve their sensor-generated health information via an Amazon Echo Show device with touch display and voice-activated capabilities, and a web-based interface. Data were collected over two years, and feedback was solicited during four quarterly interviews and one exit interview. Thematic analysis revealed participants used the Echo Show to retrieve their health information, set medication/health appointment reminders, and for non-health related purposes (e.g. music, weather). Regular retrieval of health information was low, partly due to technology skills, lack of interest, health issues, and technical/internet issues. Despite this, participants reported a sense of security in having the sensors installed and valued the depth sensor’s fall detection abilities. This study yielded many additional findings that will be presented, such as participant recommendations to facilitate greater adoption. Implications can inform technological solutions for older adult health self-management and aging-in-place.

